# Kill two birds with one stone: Engineered exosome-mediated delivery of cholesterol modified YY1-siRNA enhances chemoradiotherapy sensitivity of glioblastoma

**DOI:** 10.3389/fphar.2022.975291

**Published:** 2022-08-19

**Authors:** Xiao Liu, Zhengcong Cao, Nannan Liu, Guangxun Gao, Mingrui Du, Yingwen Wang, Boyang Cheng, Maorong Zhu, Bo Jia, Luxiang Pan, Wangqian Zhang, Yuran Jiang, Wei He, Linlin Xu, Wei Zhang, Qunxing An, Qingdong Guo, Jintao Gu

**Affiliations:** ^1^ State Key Laboratory of Cancer Biology, Biotechnology Center, School of Pharmacy, The Fourth Military Medical University, Xi’an, China; ^2^ The First Affiliated Hospital, The Fourth Military Medical University, Xi’an, China; ^3^ Experimental Teaching Center of Basic Medicine, The Fourth Military Medical University, Xi’an, China; ^4^ The Second Affiliated Hospital, The Fourth Military Medical University, Xi’an, China; ^5^ The Third Affiliated Hospital, The Forth Military Medical University, Xi’an, China

**Keywords:** glioblastoma, Yin Yang 1, T7, exosome, chemoradiotherapy sensitization

## Abstract

Glioblastoma (GBM) is the most malignant tumor of the central nervous system in adults. Irradiation (IR) and temozolomide (TMZ) play an extremely important role in the treatment of GBM. However, major impediments to effective treatment are postoperative tumor recurrence and acquired resistance to chemoradiotherapy. Our previous studies confirm that Yin Yang 1 (YY1) is highly expressed in GBM, whereby it is associated with cell dedifferentiation, survival, and therapeutic resistance. Targeted delivery of small interfering RNA (siRNA) without blood-brain barrier (BBB) restriction for eradication of GBM represents a promising approach for therapeutic interventions. In this study, we utilize the engineering technology to generate T7 peptide-decorated exosome (T7-exo). T7 is a peptide specifically binding to the transferrin receptor. T7-exo shows excellent packaging and protection of cholesterol-modified Cy3-siYY1 while quickly releasing payloads in a cytoplasmic reductive environment. The engineered exosomes T7-siYY1-exo could deliver more effciently to GBM cells both *in vitro* and *in vivo*. Notably, *in vitro* experiments demonstrate that T7-siYY1-exo can enhance chemoradiotherapy sensitivity and reverse therapeutic resistance. Moreover, T7-siYY1-exo and TMZ/IR exert synergistic anti-GBM effect and significantly improves the survival time of GBM bearing mice. Our findings indicate that T7-siYY1-exo may be a potential approach to reverse the chemoradiotherapy resistance in GBM.

## Graphical Abstract

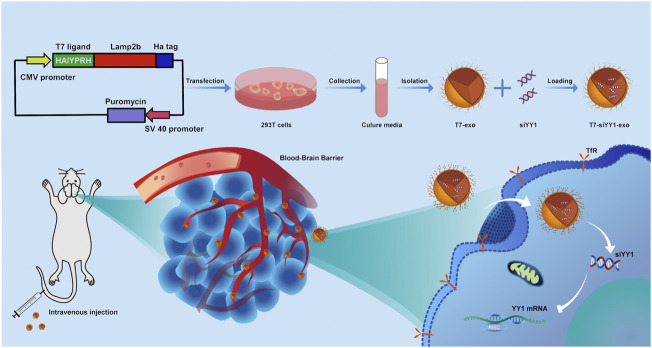

## Introduction

Glioblastoma (GBM) is a primary brain cancer with the highest mortality (Movahedpour et al., 2021). The average lifespan of patients with GBM is less than 1 year, and only 5% of them live for more than 5 years. The standard treatment for glioblastoma is surgical resection, followed by radiotherapy and chemotherapy with temozolomide (TMZ) (Kim et al., 2020). Nevertheless, surgical treatment is limited because of the high invasiveness of GBM and may not suitable for some patients depending on their condition. In addition, most drugs including TMZ are very toxic with severe side-effects (Yasaswi et al., 2021). Despite the maximal surgical resection followed by adjuvant chemoradiotherapy, the median time to tumor recurrence is approximately 8 months (Adamus et al., 2022). To overcome this problem, new strategies are necessary to counteract both TMZ and radiation resistance.

Yin Yang 1 (YY1), a transcription factor overexpressed in GBM, has been identified in our research as well as in previous research. As reported, it can help to regulate cell dedifferentiation, cell survival, and therapeutic resistance (Gu et al., 2021). Moreover, according to previous studies, knockdown of YY1 can significantly block the tumor malignance and reverse the resistance (Sarvagalla et al., 2019). Considering that YY1 overexpression is observed in many cancers and has various biological functions with respect to the hallmarks of cancer, it is an attractive approach to utilize YY1 as a novel target for therapeutic interventions. However, cancer-promoting transcription factors cannot be easily targeted due to their nuclear localization, YY1 is predominantly present in nuclei and not easily targeted (Verheul et al., 2020).

Gene therapy is a new clinical strategy for GBM treatment and has attracted extensive attention recently. Compared with other existing techniques for gene regulation, RNA interference is a methodology able to silence cancer-promoting genes as reported (Cui et al., 2018). Small interfering RNA (siRNA) combined with chemotherapy is considered as one promising strategy against cancer, and many studies demonstrated the outcomes of such co-treatment are significantly better than those using the siRNA or chemotherapeutic drug alone (Xiao et al., 2018). However, siRNA is unstable in systemic circulation and bio-membrane permeability. Thus it is necessary to find suitable carriers for the effective delivery of siRNA *in vivo* (Ruan et al., 2018). Examinations confirmed the effectiveness of liposomal, viral vectors and nanoparticles for RNA transfection, while they are immunogenic, toxic and degrade slowly (Kase et al., 2021).

Exosomes are small membrane vesicles of endocytic origin, which can be released into the extracellular environment during the fusion of multivesicular bodies with the cytomembrane (Yu et al., 2022). Because of their low immunogenicity and toxicity, biodegradability, and strong ability to protect endogenous biologically active ingredients, exosomes have become a new therapeutic strategy in drug delivery (Fu et al., 2021). However, natural exosomes lack targetability and present a rapid accumulate in peripheral organs like the spleen and liver following the systemic treatment, instead of targeting specific tissues (Jiang et al., 2018). According to recent studies, specific ligands can be expressed on the exosome membrane surface via gene modification, achieving the targeted exosome delivery (Huang et al., 2022). In brain tumor, the TfRs are highly expressed in brain microvascular endothelial cells (BMVECs) and GBM cell line for regulation of brain uptake of iron (Tortorella and Karagiannis, 2014; Choudhury et al., 2018; Mojarad-Jabali et al., 2022). T7 is a transferrin receptor (TfR)-binding peptide with the sequence HAIYPRH. On that account, it is possible that T7 is an ultrahigh-efficiency targeting strategy for GBM-targeted delivery.

Herein, a TfR-targeting exosome was produced by creating a fusion protein of T7 peptide and Lamp2b. After systemic administration, the T7 ligand-decorated exosome (T7-exo) could bind to GBM in the brain and enhance the efficiency of cholesterol-modified siYY1 delivery. Therefore, T7-exo was evaluated as a GBM-targeting carrier of siYY1. In the orthotopic GBM mice model, combined with temozolomide or radiotherapy, targeting knockdown of YY1 via cholesterol-modified siYY1 delivered by engineered exosomes synergistically inhibited the growth of GBM. Overall, T7-siYY1-exo has the potential to overcome chemoradiotherapy resistance in GBM by multiple mechanisms.

## Results

### Characterization of T7-decorated exosomes and siYY1-loaded exosomes

**FIGURE 1:**
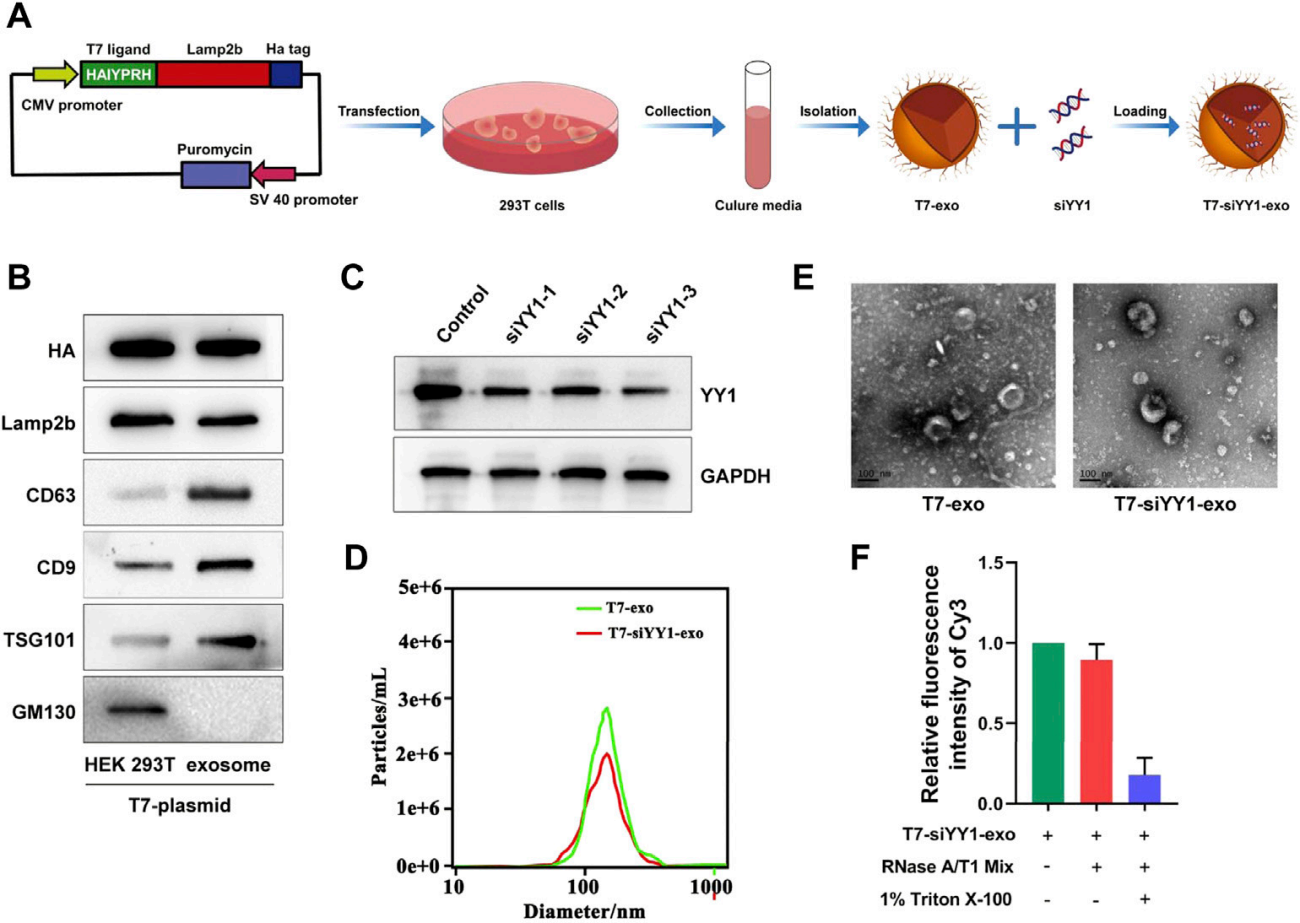
Preparation and characterization of T7-siYY1-exo. **(A)** Schematic diagram of preparation and isolation of T7-siYY1-exo. **(B)** Detection of CD9, CD63, TSG101, Lamp2b, HA, and GM130 expression by Western blot. **(C)** Detection of YY1 knockdown efficiency. **(D)** Size distribution of T7-exo and T7-siYY1-exo measured by NANO SIGHT. **(E)**Transmission electron micrograph of T7-exo and T7-siYY1-exo. **(F)** qRT-PCR analysis of siYY1-Cy3 after treatments with RNase A/T1 Mix and 1% Triton X-100 for 30 min.

To generate T7-exo, stable cell lines were prepared by transfection of plasmids encoding T7-Lamp2b into 293T cells (Figure 1A). Ligand-decorated exosomes were then isolated and confirmed by western blotting with an anti-HA-tag antibody which could be expressed as a fusion protein regarding ligand-Lamp2b. Moreover, exosome membrane proteins CD9, CD63, TSG101, and GM130 assisted in further identifying the T7-exo (Figure 1B). These data confirmed that T7-exo had characteristics of exosomes and targeting ligands. To screen out a cholesterol-modified Cy3-siYY1 sequence of which gene knockdown efficiency is best, three siRNA sequences for YY1 were designed and transfected into LN229 cells. The results showed that the third siRNA sequence exhibited a gene knockdown efficiency of more than 80%, significantly higher than the other two (Figure 1C), and the third siRNA YY1 was used for subsequent experiments. Then, T7-siYY1-exo was prepared and characterized. The isolated exosomes had a homogenous size, and a single peak was shown in distribution graphs. The dimensions of exosomes were measured at about 120 nm, and there was no significant effect on the size of exosomes encapsulated with siYY1 (Figure 1D). TEM images assisted in evaluating the morphology of exosomes and siYY1-loaded exosomes. The morphology of unmodified exosomes was not obviously different from that of ligand-decorated T7-siYY1-exo (Figure 1E). Additionally, when treated with RNase A/T1 mix, it was found that the Cy3 fluorescence intensity was not reduced, which proved that siYY1 was protected, unless 1% Triton X-100 was co-applied to the treatment, which resulted in the degradation of exosome membranes (Figure 1F). These findings revealed that the engineered exosomes were successfully isolated and prepared.

### *In vitro* BBB/BBTB penetrating ability of T7-siYY1-exo

**FIGURE 2:**
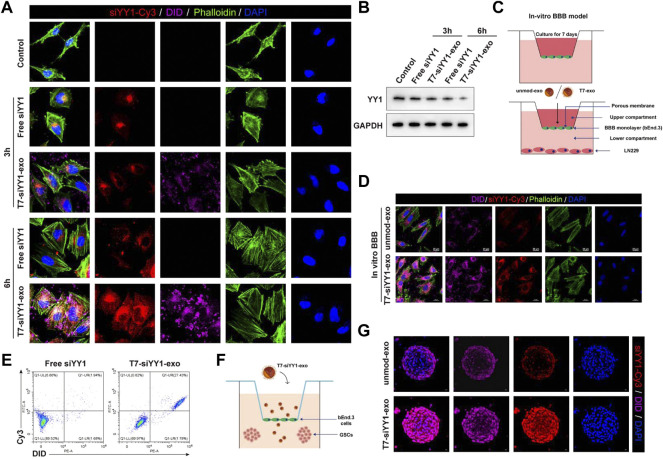
Cellular uptake of T7-siYY1-exo in vitro and BBB/BBTB model. **(A)** The delivery efficiency of Free siYY1 and T7-siYY1-exo in LN229 cells. Cy3-labeled siYY1 and loaded DID-labeled exosomes (5 μg/ml) were used to treat LN229 cells. After 24h incubation, the cytoskeleton was stained with phalloidin and the cell nuclei were stained with DAPI. The fluorescent was photographed under a confocal laser scanning microscope using Nikon NIS-Elements software (Nikon, Tokyo, Japan). **(B)** The knockdown efficiency of YY1 was detected by Western blot. **(C)** Schematic diagram of the BBB model *in-vitro*. **(D)** Representative immunofluorescence images showing T7-siYY1-exo uptake into LN229 cells after passing through a bEnd.3 monolayer. Unmod-exo/T7-siYY1-exo (5 μg/ml) was added to upper compartment. The fluorescent was photographed under a confocal laser scanning microscope using Nikon NIS-Elements software (Nikon, Tokyo, Japan). **(E)** The positive rates of DiD and Cy3 were detected by flow cytometry. **(F,G)** The penetrating and tumor targeting efficacy of T7-siYY1-exo evaluated through BBTB/LN229 tumor spheroids co-culture model. The treatment concentration of unmod-exo/T7-siYY1-exo was 5 μg/ml. The fluorescent was photographed under a confocal laser scanning microscope using Nikon NIS-Elements software (Nikon, Tokyo, Japan).

To evaluate the siYY1 delivery efficiency of T7-exo, electroporation was applied to loading the cholesterol-modified cy3-siYY1 into exosomes to form complexes. Fluorescence microscope results showed that cholesterol-modified cy3-siYY1 could be more efficiently integrated into cells via T7-exo than transfection alone to facilitate gene knockdown (Figure 2A). The T7-siYY1-exo group showed a lower YY1 expression than the Free-siYY1 group. Both Free-siYY1 and T7-siYY1-exo could silence YY1 *in vitro*, with the latter exhibiting a more substantial effect (Figure 2B).

An *in vitro* BBB model which has a transwell system was first used for assessing the ability of T7-siYY1-exo to penetrate the BBB. Under this system, bEnd.3 cells were cultured in the upper compartment and used T7-siYY1-exo and Free siYY1 for treatment, respectively, together with culturing LN229 cells in the lower room (Figure 2C). As shown in Figure 2D, only T7-siYY1-exo could pass through the BBB monolayer and reach the LN229 cells cultured in the lower compartment. Flow cytometry assisted in examining the DiD and Cy3 signal intensity, finding T7-exo significantly enhanced Cy3 signal in LN229 cells compared to Free siYY1 (Figure 2E). It can be concluded that T7-exo can penetrate the *in vitro* BBB model meanwhile accumulating in the cytoplasm of LN229 cells.

To evaluate the transcytosis efficiency of T7-siYY1-exo, we established the bEnd.3/LN229 tumor spheroids co-culture model for imitating the blood−brain tumor barrier (BBTB) *in vitro* (Figure 2F). Interestingly, the Cy3 and DiD fluorescence were powerful inside the tumor spheroids receiving the treatment with T7-siYY1-exo, supporting T7-siYY1-exo effectively penetrated into GBM. In contrast, tumor spheroid receiving the treatment of unmod-exo displayed weak Cy3 and DiD fluorescence only in the superficial area (Figure 2G). The above findings confirmed that the functionalization of the exosome with T7 peptide is capable of remarkably enhancing the BBTB penetration ability and the GBM targeting ability.

### T7-siYY1-exo enhanced TMZ and radiation sensitivity of GBM cells *in vitro*

**FIGURE 3:**
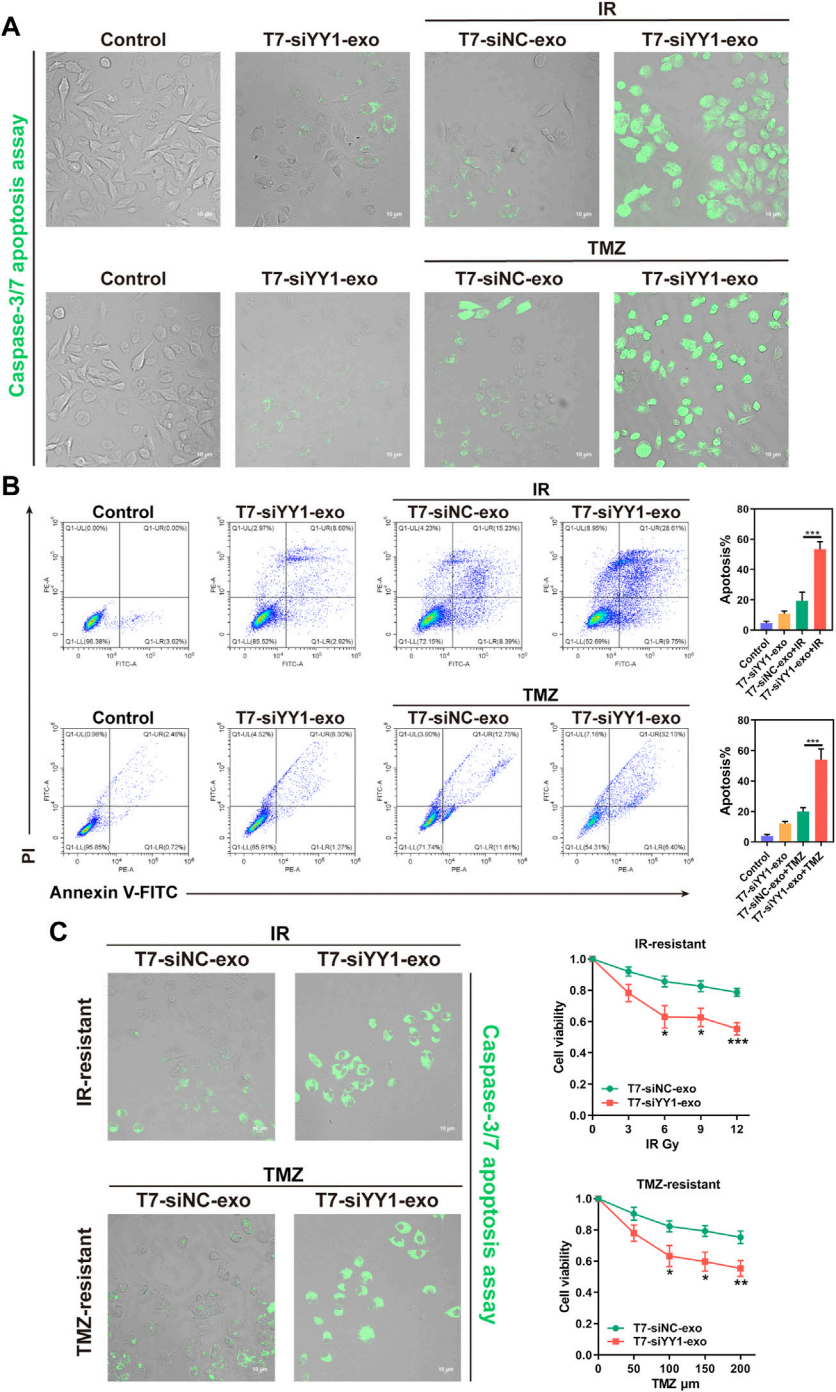
The therapeutic effect of T7-siYY1-exo in vitro. **(A)** LN229 cells were cultured in the presence of T7-siNC-exo/T7-siYY1-exo (5 μg/ml) and TMZ (50 μM)/IR (6Gy) for 2 days and apoptotic cells were visualized by caspase-3/7 apoptosis assay. The fluorescent was photographed under a confocal laser scanning microscope using Nikon NIS-Elements software (Nikon, Tokyo, Japan). Data in a are representative of three independent biological experiments. **(B)** LN229 cells administrated with T7-siNC-exo/T7-siYY1-exo (5 μg/ml) and TMZ (50 μM)/IR (6Gy) were subjected to FACS to detect apoptosis. *p* values are displayed as *p ≤ 0.05, **p ≤ 0.01, ***p ≤ 0.001. **(C)** The effect of T7-siYY1-exo on LN229-IR-resistant and LN229-TMZ-resistant cells. The concentration of TMZ was 200 μM and the dose of IR was 12Gy. *p* values are displayed as *p ≤ 0.05, **p ≤ 0.01, ***p ≤ 0.001.

The above findings revealed the effective taking up of engineered exosomes by recipient cells. We then determined whether T7-siYY1-exo was sufficient to enhance the chemoradiotherapy sensitivity of GBM cells. Apoptotic cells were visualized by caspase-3/7 apoptosis assay. The single delivery of siYY1 could not bring perfect and effective treatment, indicating that single siYY1 lacked strong therapeutic function. Besides, T7-siYY1-exo together with TMZ or IR led to a substantial enhancement of apoptosis compared with that of the cells treated with T7-Negative Control of siRNA-exo (T7-siNC-exo) together with TMZ or IR (Figure 3A). Flow cytometry analysis of LN229 cells undergoing apoptosis found that T7-siYY1-exo caused a noticeable increase in TMZ and radiation sensitivity. That is to say, T7-siYY1-exo exhibited an enhanced synergistic effect on inducing apoptosis in the target cells (Figure 3B).

In order to further observe the sensitizing effect of T7-siYY1-exo on chemoradiotherapy resistant cells, a TMZ-resistant derivative of the LN229 GBM cell line (LN229-TMZ-resistant) was produced via the serial passage of these cells in the presence of increasing TMZ concentrations. Moreover, LN229-IR-resistant cells were generated by parental cells after 6 times of 2Gy irradiation. Surprisingly, T7-siYY1-exo enhanced the sensitivity of LN229-TMZ-resistant and LN229-IR-resistant cells to chemotherapy and radiotherapy, reversed the therapeutic resistance of cells (Figure 3C). Thus, the results suggested that T7-siYY1-exo can simultaneously enhance the sensitivity of GBM cells to chemotherapy and radiotherapy.

**FIGURE 4:**
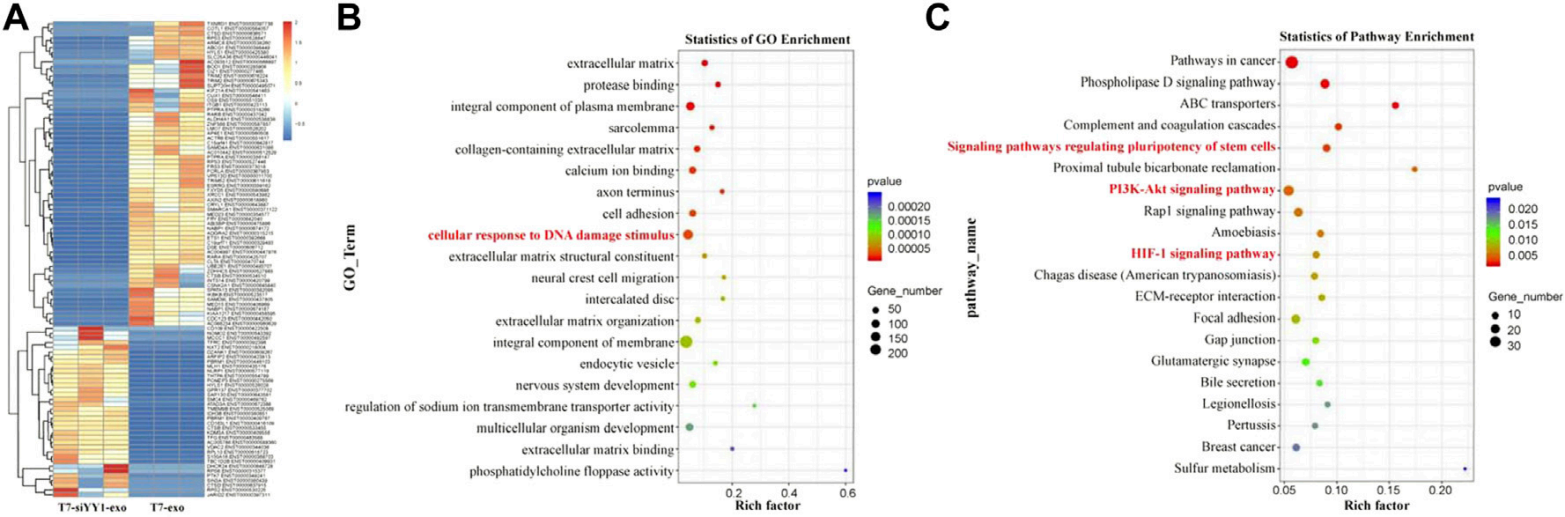
Molecular mechanism of T7-siYY1-exo. **(A)** RNA sequencing was performed on T7-siYY1-exo treated cells. **(B)** Gene ontology (GO) enrichment analysis enrichment of differential genes after T7-siYY1-exo treatment. **(C)** KEGG bioinformatics analysis of differential genes after T7-siYY1-exo treatment.

To gain insights into the mechanism of T7-siYY1-exo, transcriptomic sequencing was performed on T7-siYY1-exo and T7-exo treated cells (Figure 4A). Gene ontology (GO) analysis showed that gene sets for cellular response to DNA damage stimulus were enriched in T7-siYY1-exo treatment cells (Figure 4B). Additionally, KEGG bioinformatics analysis revealed the enrichment of signaling pathways that regulated the pluripotency of stem cells, PI3K-Akt signaling pathway and HIF-1 signaling pathway in T7-siYY1-exo treatment cells (Figure 4C). Indeed, previous studies revealed the close association of these gene sets with TMZ and radiotherapy resistance (Persano et al., 2012; Tomar et al., 2020; Nie et al., 2021).

### Efficiency of *in vivo* systemic delivery of T7-exo to the brain tumor

**FIGURE 5:**
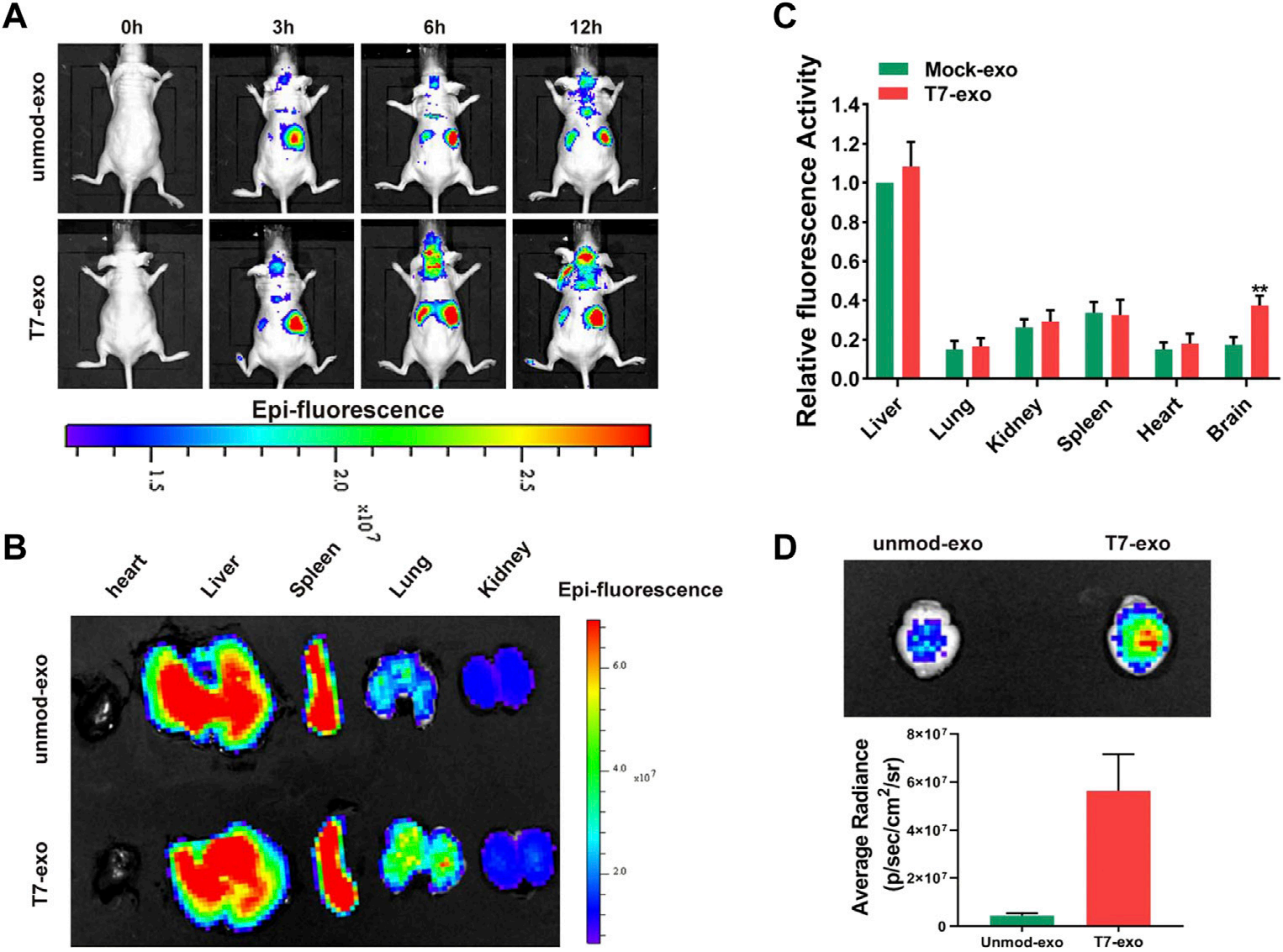
Tumor targeting ability of T7-siYY1-exo in orthotopic GBM xenograft model using LN229 cells. **(A)** *In vivo* florescence imaging of Orthotopic GBM xenograft mice at 0, 3, 6 and 12 h time point after intravenous administration of unmod-exo/T7-siYY1-exo (200 ug). **(B)** *Ex vivo* fluorescence images of Liver, Spleen, Lung, Heart and Kidney from mice sacrificed at 12 h post-injection. **(C)** Fluorescence quantitative analysis of *ex vivo* organs of LN229 tumor-bearing mice after intravenous injection. *p* values are displayed as *p ≤ 0.05, **p ≤ 0.01, ***p ≤ 0.001. **(D)** *Ex vivo* images and quantitative analysis of the GBM-bearing brains.

The uptake and the function of T7-exo were identified in GBM cells *in vitro*, followed by the determination of the biodistribution of T7-exo *in vivo*. We injected 200 μg of DiD-labeled unmod-exo or T7-exo loaded with 0.5OD Cy3 labeled siYY1 into mice via the tail vein, and detect fluorescence signal through *in vivo* imaging system (IVIS). As shown in Figure 5A, the intensity and distribution of fluorescence were recorded at different time points (0, 3, 6, 12 h) after mock unmod-exo or T7-exo injection. According to the fluorescence quantification of different organs (brain, heart, liver, spleen, lung, and kidney), we found that the fluorescence intensity exhibited an obvious concentration in the peripheral organs at 12 h following the injection with unmod-exo or T7-exo. Then, the assessment on the ability of T7-exo to deliver cholesterol-modified siYY1 *in vivo* was conducted via Cy3 fluorescence intensity, detecting the relative fluorescence intensity of siYY1-Cy3 in various organs of mice after unmod-exo or T7-exo injection. As shown in Figure 5B, siYY1-Cy3 exhibited enrichment in a variety of organs at different time points of post-injection. Unsurprisingly, fluorescent images of the other organs show that T7-siYY1-exo was mainly metabolized in the liver (Figure 5C). Notably, based on the *ex vivo* imaging regarding brains at 12 h together with the fluorescence quantitative analysis, the T7-siYY1-exo showed an obvious accumulation in GBM tissue instead of normal brain tissue (Figure 5D). These results revealed that T7-modified exosomes possessed a better ability to overcome BBB and target GBM than did unmodified exosomes.

### T7-siYY1-exo enhance the *in vivo* efficacy of chemotherapy and radiotherapy for GBM

**FIGURE 6:**
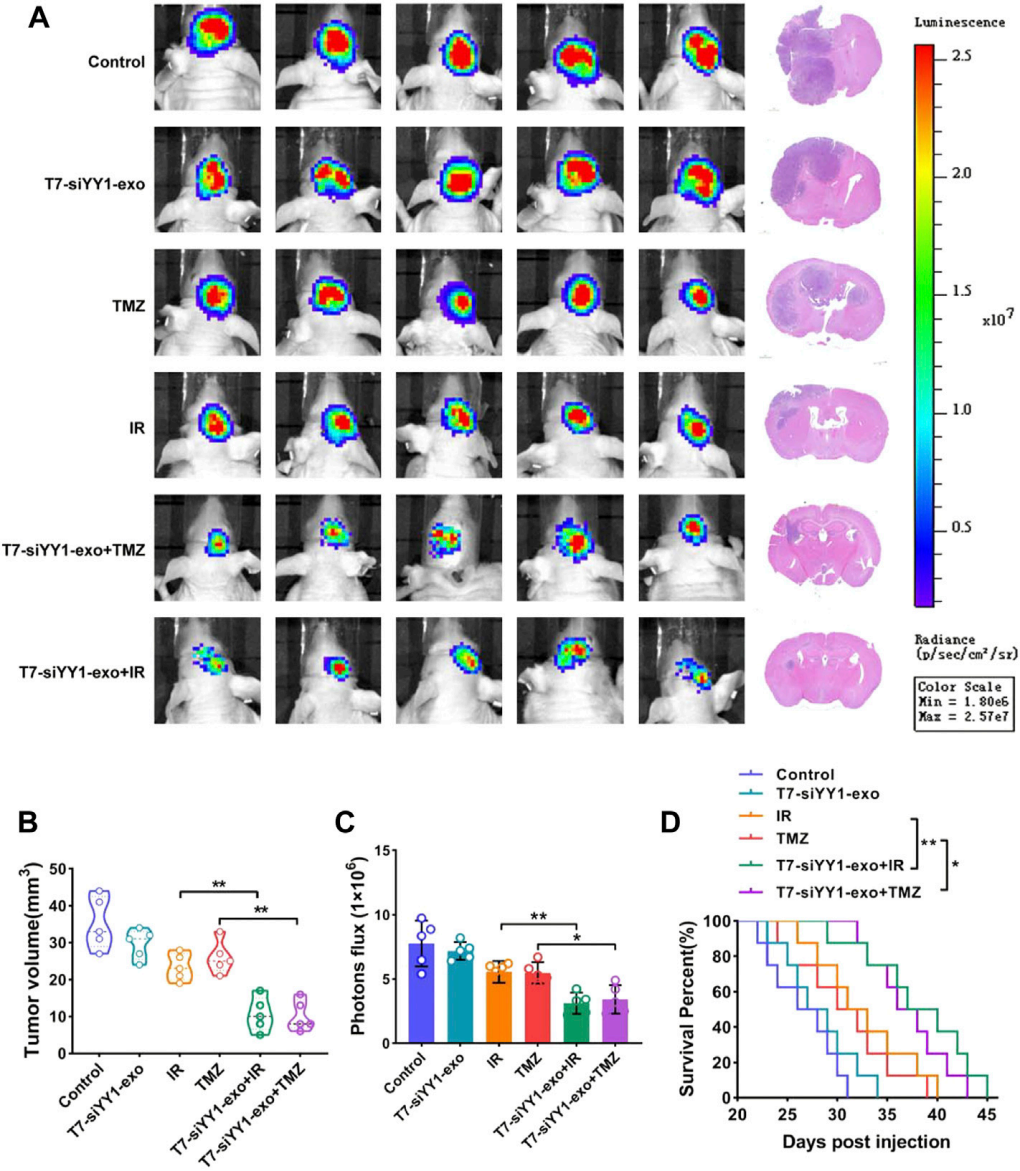
Anti-tumor efficiency of T7-siYY1-exo in vivo. **(A)** Left, representative images of *in vivo* orthotopic GBM bioluminescence. Right, Representative images of H&E staining. Bioluminescence images were captured by IVIS on day 21 in all groups. After mice sacrifice, brain tissue samples were obtained for HE staining. **(B)** The tumor volume was calculated using a modified ellipsoid formula. Brain tissue samples were obtained after mice sacrifice. *p* values are displayed as *p ≤ 0.05, **p ≤ 0.01, ***p ≤ 0.001. **(C)** Quantification of the bioluminescence of xenografts derived from the luciferase-labeled LN229 cells in each group. Bioluminescence images were captured by IVIS on day 21 in all groups. *p* values are displayed as *p ≤ 0.05, **p ≤ 0.01, ***p ≤ 0.001. **(D)** Kaplan–Meier survival plot was graphed to evaluate mice lifespan in each group, mice were collected at end stage. *p* values are displayed as *p ≤ 0.05, **p ≤ 0.01, ***p ≤ 0.001.

To investigate the *in vivo* effects of T7-siYY1-exo for GBM, we established Orthotopic GBM xenograft model as described. Tumors were allowed for a 14 days growth followed by treatment daily with saline, T7-siYY1-exo, IR, TMZ, IR + T7-siYY1-exo or TMZ + T7-siYY1-exo for 7 days, and tumor signals were evaluated using luciferase bioluminescence. Based on the tumor bioluminescence quantification, T7-siYY1-exo combined with TMZ or IR could hinder the tumor growth to a much larger extent than TMZ or IR alone (Figure 6A). It should further be noted that T7-siYY1-exo monotherapy had a slightly treatment effect on GBM. The tumor volume as a reflection of tumor burden, Hematoxylin-eosin (HE) staining showed that T7-siYY1-exo combined with TMZ or IR regimens showed significantly increased antitumor activity, relative to monotherapy (Figures 6B,C). For further examining the efficacy of T7-siYY1-exo against GBM *in vivo*, we plotted as well as monitored Kaplan-Meier survival curves regarding the model mice, finding that relative to mice in the control group, treatment with IR and TMZ helped to extend a little the survival of GBM mice. Importantly, mice administrated with T7-siYY1-exo and TMZ/IR had an obvious loner survival time compared to mice receiving IR and TMZ alone (Figure 6D).

### *In vivo* safety evaluation

**FIGURE 7:**
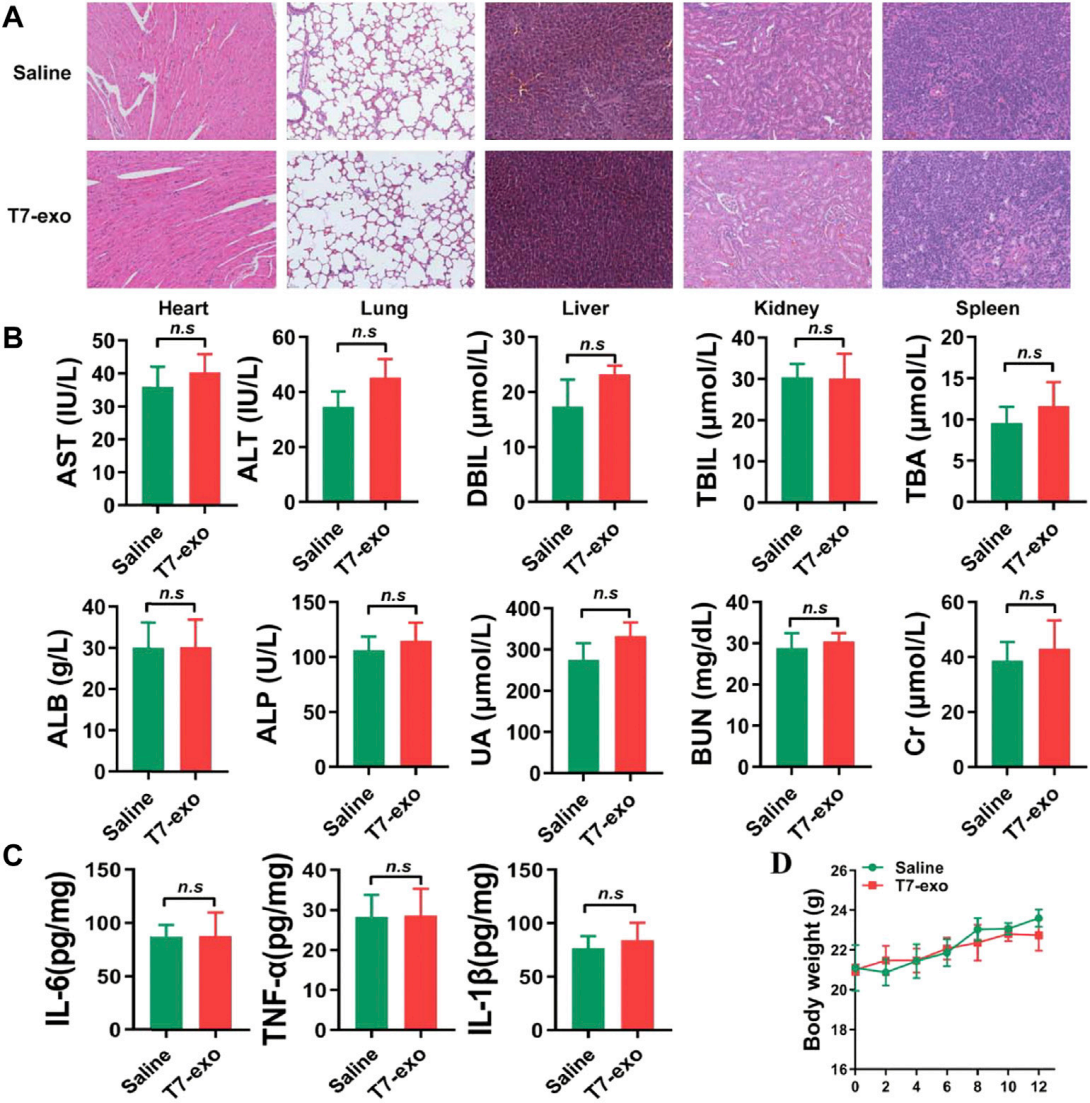
The systematic toxicity assessment of T7-siYY1-exo. **(A)** Histological analyses of liver, heart, kidney, lung and spleen sections stained with H&E of BALB/c mice post-intravenous injection of T7-siYY1-exo (200 μg) or Saline for 12 days (one dose every other day). **(B)** Clinical chemistry and hematology parameters for T7-siYY1-exo treated mice. *p* values are displayed as not significant (ns) for *p* > 0.05. **(C)** The expression level of serum inflammatory cytokines TNF-α, IL-1β and IL-6. **(D)** Body weight changes in T7-siYY1-exo treated mice.

Toxicity is considered to be another significant parameter of a suitable delivery vehicle besides the delivery efficiency. In the *in vivo* safety experiment, in order to eliminate the influence of immune deficiency, we carried out relevant experiments in BALB/c mice. We intravenously injected 15 mg/kg T7-exo into healthy BALB/c mice for 12 days for evaluating the systematic toxicity exhibited by T7-exo. Obviously, the mononuclear phagocyte system absorbed as well as cleared the majority of exosomes after intravenous injection. Hence, we conducted an investigation on the T7-exo-induced potential pathological damage on such organs. In the group receiving T7-exo treatment, major tissues, including the heart, liver, spleen, lung, and kidney, did not exhibit any distinct histopathological abnormalities or damage (Figure 7A). Blood biochemistry together with the hematology analysis assisted in revealing the potential toxic action exerted by exosomes over treated mice. Measurement of the key biomarkers of liver and kidney injury, including alanine aminotransferase (ALT), aspartate aminotransferase (AST), alkaline phosphatase (ALP), direct bilirubin (DBIL), total bilirubin (TBIL), total bile acid (TBA), serum albumin (ALB), creatinine (CR), uric acid (UA) and blood urea nitrogen (BUN) was performed before the completion of the experiment. As shown in Figure 7B, all the above indicators were the same as animals treated with saline, indicating that T7-exo had no significant hepatorenal toxicity within the dosing regimen. No obvious immune response was elicited in T7-exo and saline, as serum inflammatory cytokines like IL-1β, TNF-α and IL-6 exhibited similar levels to the saline control group (Figure 7C), suggesting that T7-exo did not lead to an inflammatory response. During the study period, the experimental group presented no deaths or serious weight loss (Figure 7D). These results showed that T7-exo did not cause acute toxicity to the hematological system and major organs in mice.

## Materials and methods

### Cell culture and treatment

Human GBM cell lines LN229 were obtained from American Type Culture Collection (ATCC). The cell line bEnd.3 was acquired from Beina Chuanglian Biology Research Institute (Beijing, China). All cells were cultured in Dulbecco’s modified Eagle’s medium (DMEM) supplemented with 10% FBS (Gibco, Australia origin), 1% penicillin/streptomycin (NCM biotech, Suzhou, China) at 37°C with 5% CO_2_.

### Establishment of the TMZ-resistant cell line

The LN229 cells were inducted of drug resistance by TMZ (TOPSCIENCE, Shanghai, China) with increasing concentration gradient as following concentrations: 20, 50, 100, 200, 400 μM. Each dose was maintained for 15 days, and finally a stable TMZ-resistant cell line was obtained. Simultaneously, intermittent high-concentration stimulation (800 μM TMZ for 48 h) and maintenance was conducted as necessary.

### pcDNA3.1 (+)-T7 ligand-LAMP2B-HA construction, preparation

For construction of a T7-Lamp2b plasmid (pT7-Lamp2b), the cDNA fragments encoding the T7 (HAIYPRH) peptide and Lamp2b were inserted downstream of the CMV promoter in the pcDNA3.1 vector. The Human influenza hemagglutinin (HA) tag was added at the end of the Lamp2b sequence.

### Preparation of ligand-decorated exosomes

To prepare stable cell lines for generation of ligand-decorated exosomes, 293T cells were cultured in DMEM supplemented with 10% FBS. T7-Lamp2b were transfected into the cells using Lipo 3,000 according to the manufacturer’s manual. After 4 h of transfection, the medium was replaced with fresh medium containing puromycin at 2 μg/ml concentration. Stable 293T cell lines containing T7-Lamp2b were cultured for 3 days in DMEM supplemented with 10% FBS. Then, the medium was harvested, the cell medium containing exosomes was harvested by centrifugation at 300 *g* for 5 min to eliminate cells and subsequently centrifuged at 10,000×g for 30 min to remove dead cells and cell debris. Finally, the clear supernatant was centrifuged for 90 min at 120,000×g to pellet exosomes using ultracentrifuge (Beckman, United States). At last, exosomes were resuspended into 100 μL of 1× PBS and stored immediately at - 80°C. All the centrifugation steps were carried out at 4°C.

### Nanoparticle tracking analysis

The NTA was performed as described previously (Mao et al., 2017). In brief, the size distribution of the exosomes was analyzed using a Nano Sight LM10 instrument (Marvern instruments, UK). The particle suspensions were diluted with PBS to a concentration of 10^8^ particles/mL for analysis.

### Screening of siRNA sequences

To screen the effective cholesterol-modified siYY1 sequence that silenced YY1 protein, we designed three different siRNA sequences and transfected into LN229 (Table 1). The YY1 protein was collected and examined by Western Blot to screen the sequence with the highest silence efficiency.

**TABLE 1:** The siRNA sequence of YY1.

siRNA constructs	Target sequence in mRNA (5′-3′)
YY1 siRNA-1 sense	CGA​CGA​CUA​CAU​UGA​ACA​ATT
YY1 siRNA-1 antisense	UUG​UUC​AAU​GUA​GUC​GUC​GTT
YY1 siRNA-2 sense	CGA​CGA​CGA​CUA​CAU​UGA​ATT
YY1 siRNA-2 antisense	UUC​AAU​GUA​GUC​GUC​GUC​GTT
YY1 siRNA-3 sense	GAU​GAU​GCU​CCA​AGA​ACA​ATT
YY1 siRNA-3 antisense	UUG​UUC​UUG​GAG​CAU​CAU​CTT

### Loading of siYY1 oligonucleotide into exosomes

The cholesterol-modified siYY1 sequence of YY1 is 5′- GAU​GAU​GCU​CCA​AGA​ACA​ATT-3′; 5′-UUG​UUC​UUG​GAG​CAU​CAU​CTT-3′. To encapsulate siYY1 into exosomes, exosomes at a total protein concentration of 20 µg were mixed with 20 µg of siYY1 in 400 µL of PBS. The mixture was electroporated at 400 V in a 4 mm cuvette. Unloaded siYY1 were removed by ultracentrifugation at 120,000 g. The supernatants were removed and the pellets were resuspended.

### *In vitro* BBB model

An *in-vitro* BBB model was established using LN229 and bEnd.3 cells as described previously (Yang et al., 2021). In brief, bEnd.3 cells were seeded into the upper chamber of Transwell inserts coated with 2% gelatin solution and cultured for 7 days in DMEM supplemented with 10% FBS. LN229 cells were seeded in the lower Transwell compartment. Cy3-labeled siYY1 and T7-siYY1-exo were added to the upper chamber to assess the penetration efficiency of free siYY1 and T7-siYY1-exo across the BBB.

### Exosomes labeling

The fuorescent dye DiD was purchased from Lumiprobe (United States) and used to label exosomes. Purified exosomes were incubated in the presence of 5 mM DiD for 15 min at 37°C then ultracentrifuged at 120,000×g, 90 min to remove the unbounded dye. After being washed twice in PBS with 120,000×g centrifugation, the labeled exosomes were resuspended in PBS prior to use.

### RNA protection assay

T7-siYY1-exo were incubated with 20 μL RNase A/T1 mix with or without 1% Triton X-100 at 37°C for 30 min. The fluorescence intensity of Cy3 was detected by confocal microscope.

### Cell viability assay

For the cell viability assay, 5×10^3^ cells were plated in 96-well plates and treated in triplicate for 3 days with TMZ. Cell proliferation was estimated using the Cell Counting Kit-8 (CCK-8) following the protocol, the absorbance was measured at 450 nm using a Microplate Reader (Biotek, United States).

### Orthotopic mouse xenografts

Male, 6-week-old BAL B/c nude mice (∼18 g) were obtained from Gempharmatech Co., Ltd. (Nanjing, China). GBM cells expressing luciferase were intracranially transplanted into immunocompromised mice. In brief, a burr hole was made 2.5 mm left of the sagittal suture and 0.5 mm anterior to the bregma using a dental drill with a diameter of 0.7 mm, and the injection depth is 2.5 mm. To examine the tumor growth, animals were administrated intraperitoneally with 3.0 mg/100 uL solution of D-Luciferin potassium salt (Abcam, United States) and anesthetized with isoflurane for the imaging analysis. The tumor luciferase images were captured by using an IVIS 100 imaging system (PerkinElmer, United States).

### Animal studies

For *in vivo* evaluating delivery of engineered T7-exos, unmod-exos and T7-exos (about 200 ug) were labeled with the DID and injected into the tumor-bearing mice via the tail vein to analyze the distribution of exosomes. Fluorescence signals were detected by IVIS at 0, 3, 6 and 12 h after injection. Afterwards, the mice were sacrificed by cervical dislocation, then the organs and tumors were taken out, and the distribution of fluorescently labeled exosomes in various organs was observed using IVIS.

To evaluate *in vivo* effect of T7 exos, orthotopic mice xenografts with Luc-LN229 cells were divided into six groups. Saline, T7-siYY1-exo, TMZ, IR, T7-siYY1-exo + TMZ and T7-siYY1-exo + IR. The treatment was started after tumor formation on day 14. All animal studies were performed in accordance with protocols approved by the Ethical Committee and Institutional Review Board of Fourth Military Medical University.

### *In vivo* safety evaluation

Eight female BALB/c mice were randomly divided into two groups. One group received an intravenous injection of T7-siYY1-exo (20 mg/kg) at one dose for 12 days and the other group was treated with Saline as control. Blood samples and major organ tissues were collected at 24 h after the last administration, for hematologic and histochemistry analysis. The serum alanine aminotransferase (ALT), aspartate aminotransferase (AST), alkaline phosphatase (ALP), direct bilirubin (DBIL), total bilirubin (TBIL), total bile acid (TBA), serum albumin (ALB), creatinine (CR), uric acid (UA) and blood ureanitrogen (BUN) levels were analyzed by Hitachi 7,080 Chemistry Analyzer. Major organs such as brain, heart, lung, liver, spleen, and kidney were fixed with paraformaldehyde for 48 h and embedded in paraffin. All animal procedures were conducted in accordance with the care and use of laboratory animals’ protocol approved by the institutional animal care committee of FMMU.

### RNA preparation and qRT-PCR

Cells were harvested in Trizol (Thermo Fisher Scientific), and the total RNA was extracted using chloroform extraction and isopropanol precipitation. After spectrophotometric quantification, RNA (500 ng) was reverse transcribed into cDNA following the protocol of the PrimeScript RT Master Mix (Takara, China). The real-time PCR analyses were performed using SYBR Premix Ex Taq II on the 7,500 fast Real-Time PCR System (Applied Biosystems, United States) and Ct thresholds were determined by the matched software. Target gene expression was calculated using the 2^−ΔΔCT^ method. Each quantitative PCR assay was performed in triplicate and independently repeated three times.

### Western blot

Proteins were extracted from treated GBM cells with RIPA buffer containing proteinase inhibitor (NCM). Protein concentrations were determined by BSA protein assay kit (Thermo Fisher Scientific). Equivalent amounts of protein were separated by SDS-PAGE and transferred onto polyvinylidene fluoride membranes (PVDF). After blocking with 5% non-fat milk, membranes were successively incubated with primary (anti-HA, anti-CD63, anti-CD9, anti-GM130, anti-YY1, anti-TSG101, anti-Lamp2b anti-GAPDH) and HRP-conjugated secondary antibodies before visualizing bands using chemiluminescence (Tanon Science & Technology, Shanghai, China). The results were visualized on Tanon-5200 Chemiluminescent Imaging System (Tanon Science & Technology).

### Apoptosis assay

A FACSCalibur Flow Cytometer (BD Biosciences, United States) was used for the apoptosis assay followed the product manual. Cells were seeded in 6-well plates, harvesting in centrifuge tube after different treatment for 24h. Then washing, resuspension and staining were in accordance with the BD Pharmingen™ FITC Annexin V Apoptosis Detection Kit I (BD) protocol. The data were then analyzed with Flow Jo 10.0 software (Tree Star, San Francisco, CA, United States). Each assay was independently repeated three times.

### Statistical analysis

Data are presented as the mean ± standard deviation. One-way analysis of variance was used to determine significance among groups. Kaplan-Meier analysis was used to determine the survival curve, in which the log-rank (Mantel-Cox) test was applied to confirm significance between different groups. A value of *p* < 0.05 was considered to be significant.

## Discussion

Glioblastoma (GBM) exhibits heterogeneity, vigorous motility and large enormous ability, which is highly resistant to the existing conventional treatments, making it a kind of brain tumor with the heaviest invasiveness and fatality (Shi et al., 2018). Bevacizumab, a humanised monoclonal antibody against VEGF, received accelerated approval from the FDA for treatment of recurrent GBM, which is also used for adjuvant treatment (Tamura et al., 2019). Nevertheless, the therapeutic effect of bevacizumab combined with chemotherapy and radiotherapy or not cannot last a long time and tumors will recur 3–5 months later (Bao et al., 2021). One way to further improve the efficacy of routine treatments might be to identify genes responsible for tumor resistance. Recently, we and others demonstrated the role of YY1 on GBM cells and glioma stem cells. YY1 activates the expression of many oncogenes that affect different cellular functions, like the proliferation, the redox homeostasis, the DNA damage response, the apoptosis, the angiogenesis, the metastasis, as well as the immunosuppression (Li et al., 2020). YY1 is an attractive drug target due to its central role in tumor progression. However, it is not easy to target YY1 considering the nuclear localization.

The delivery of RNA molecules, like small interfering RNA (siRNA), microRNA (miRNA), short hairpin RNA (shRNA), as well as long non-coding RNA (lncRNA) for silencing aberrant gene expression in a cell can effectively assist in treating various transcription factors (Yang et al., 2022). Nevertheless, plenty of evidence finds that the efficacy and specificity exhibited by these therapies are not acceptable. Accumulating evidence reported that exosomes released from somatic cells might serve as proper nanocarriers of therapeutic agents specific to clinical therapeutic drugs (Shi et al., 2019). However, the way to enable exosomes to be capable of specifically delivering therapeutic molecules to target cells remains unclear. Murine pancreatic cancer cells saw the functional modification of exosomes that carry a specific siRNA for targeting oncogenic KRAS, explaining the potential of these exosomes for treating the malignant tumors with identified molecular targets (Xie et al., 2020). Our previous also found that TfR was rarely expressed in normal human astrocytes cells but significantly increased in radioresistant cells and glioma stem cells (Gu et al., 2021). Besides, a mass of TfRs is also expressed in brain capillary endothelial cells, assisting exosomes in penetrating the BBB more efficiently (Zhao et al., 2016). Kim et al. (2020) designed exosomes containing antisense microRNA oligonucleotides and decorated them with the T7-peptide. The authors aimed to selectively deliver antisense oligonucleotide against miR-21 that is commonly upregulated in GBM and involved in the inhibition of tumor cell death and consequent tumor progression.

The study of the *in vitro* and *in vivo* uptake was consistent with the proposed hypothesis that T7 functionalization promoted exosome uptake by both cerebral vascular endothelial cells and glioblastoma cells. Despite the enhanced brain targeting due to T7-modified exosomes, our study found that exosomes were remarkably enriched in peripheral organs, especially in the liver, which is consistent with previous studies. In our study, T7-siYY1-exo was used in combination with TMZ and radiotherapy, which played a better anticancer and apoptotic role in comparison with a single therapeutic strategy.

Notably, according to the histological analyses regarding major organs, the tissues of experimental mice were not damaged obviously, which indicated the favorable biocompatibility of the modified exosome. Based on these advantages, exosomes have shown great value in nucleic acid delivery, and can protect therapeutic substances from degradation and clearance by the host immune system. We believe that this modified exosome may emerge as a promising strategy for synergistic and targeted chemoradiotherapy to eradicate glioblastoma. However, it is important to emphasize that the clinical therapeutic potential of engineered exosomes is limited by the large-scale production of exosomes for clinical trials. Furthermore, the low loading efficiency of current exosome-nucleic acid-loading strategies, including electroporation, incubation, and transfection, limits their application.

## Data Availability

The original contributions presented in the study are included in the article/supplementary materials, further inquiries can be directed to the corresponding authors.

## References

[B1] Adamus T. Hung C-Y. Yu C. Kang E. Hammad M. Flores L. (2022). Glioma-targeted delivery of exosome-encapsulated antisense oligonucleotides using neural stem cells. Mol. Ther. Nucleic Acids 27, 611–620. 10.1016/j.omtn.2021.12.029 35036069 PMC8752899

[B2] Bao Z. Wang Y. Wang Q. Fang S. Shan X. Wang J. (2021). Intratumor heterogeneity, microenvironment, and mechanisms of drug resistance in glioma recurrence and evolution. Front. Med. 15 (4), 551–561. 10.1007/s11684-020-0760-2 33893983

[B3] Choudhury H. Pandey M. Chin P. X. Phang Y. L. Cheah J. Y. Ooi S. C. (2018). Transferrin receptors-targeting nanocarriers for efficient targeted delivery and transcytosis of drugs into the brain tumors: A review of recent advancements and emerging trends. Drug Deliv. Transl. Res. 8 (5), 1545–1563. 10.1007/s13346-018-0552-2 29916012

[B4] Cui X. Sun Y. Shen M. Song K. Yin X. Di W. (2018). Enhanced chemotherapeutic efficacy of paclitaxel nanoparticles Co-delivered with MicroRNA-7 by inhibiting paclitaxel-induced EGFR/ERK pathway activation for ovarian cancer therapy. ACS Appl. Mat. Interfaces 10 (9), 7821–7831. 10.1021/acsami.7b19183 29411964

[B5] Fu Z. Zhang X. Zhou X. Ur-Rehman U. Yu M. Liang H. (2021). *In vivo* self-assembled small RNAs as a new generation of RNAi therapeutics. Cell Res. 31 (6), 631–648. 10.1038/s41422-021-00491-z 33782530 PMC8169669

[B6] Gu J. Mu N. Jia B. Guo Q. Pan L. Zhu M. (2021). Targeting radiation-tolerant persister cells as a strategy for inhibiting radioresistance and recurrence in glioblastoma. Neuro. Oncol. 24, 1056–1070. 10.1093/neuonc/noab288 PMC9248405 34905060

[B7] Huang X. Wu W. Jing D. Yang L. Guo H. Wang L. (2022). Engineered exosome as targeted lncRNA MEG3 delivery vehicles for osteosarcoma therapy. J. Control. Release 343, 107–117. 10.1016/j.jconrel.2022.01.026 35077741

[B8] Jiang Y. Zhang J. Meng F. Zhong Z. (2018). Apolipoprotein E peptide-directed chimeric polymersomes mediate an ultrahigh-efficiency targeted protein therapy for glioblastoma. ACS Nano 12 (11), 11070–11079. 10.1021/acsnano.8b05265 30395440

[B9] Kase Y. Uzawa K. Wagai S. Yoshimura S. Yamamoto J. I. Toeda Y. (2021). Engineered exosomes delivering specific tumor-suppressive RNAi attenuate oral cancer progression. Sci. Rep. 11 (1), 5897. 10.1038/s41598-021-85242-1 33723306 PMC7960743

[B10] Kim G. Kim M. Lee Y. Byun J. W. Hwang D. W. Lee M. (2020). Systemic delivery of microRNA-21 antisense oligonucleotides to the brain using T7-peptide decorated exosomes. J. Control. Release 317, 273–281. 10.1016/j.jconrel.2019.11.009 31730913

[B11] Li H. Li T. Huang D. Zhang P. (2020). Long noncoding RNA SNHG17 induced by YY1 facilitates the glioma progression through targeting miR-506-3p/CTNNB1 axis to activate Wnt/β-catenin signaling pathway. Cancer Cell Int. 20 (1), 29. 10.1186/s12935-019-1088-3 32009853 PMC6988207

[B12] Mao J. Ran D. Xie C. Shen Q. Wang S. Lu W. (2017). EGFR/EGFRvIII dual-targeting peptide-mediated drug delivery for enhanced glioma therapy. ACS Appl. Mat. Interfaces 9 (29), 24462–24475. 10.1021/acsami.7b05617 28685576

[B13] Mojarad-Jabali S. Mahdinloo S. Farshbaf M. Sarfraz M. Fatahi Y. Atyabi F. (2022). Transferrin receptor-mediated liposomal drug delivery: Recent trends in targeted therapy of cancer. Expert Opin. Drug Deliv. 19 (6), 685–705. 10.1080/17425247.2022.2083106 35698794

[B14] Movahedpour A. Khatami S. H. Khorsand M. Salehi M. Savardashtaki A. Mirmajidi S. H. (2021). Exosomal noncoding RNAs: Key players in glioblastoma drug resistance. Mol. Cell. Biochem. 476 (11), 4081–4092. 10.1007/s11010-021-04221-2 34273059

[B15] Nie E. Jin X. Miao F. Yu T. Zhi T. Shi Z. (2021). TGF-β1 modulates temozolomide resistance in glioblastoma via altered microRNA processing and elevated MGMT. Neuro. Oncol. 23 (3), 435–446. 10.1093/neuonc/noaa198 32813021 PMC7992894

[B16] Persano L. Pistollato F. Rampazzo E. DellA PuppA A. Abbadi S. Frasson C. (2012). BMP2 sensitizes glioblastoma stem-like cells to Temozolomide by affecting HIF-1α stability and MGMT expression. Cell Death Dis. 3, e412. 10.1038/cddis.2012.153 23076220 PMC3481140

[B17] Ruan H. Chai Z. Shen Q. Chen X. Su B. Xie C. (2018). A novel peptide ligand RAP12 of LRP1 for glioma targeted drug delivery. J. Control. Release 279, 306–315. 10.1016/j.jconrel.2018.04.035 29679668

[B18] Sarvagalla S. Kolapalli S. P. Vallabhapurapu S. (2019). The two sides of YY1 in cancer: A friend and a foe. Front. Oncol. 9, 1230. 10.3389/fonc.2019.01230 31824839 PMC6879672

[B19] Shi S. Fu W. Lin S. Tian T. Li S. Shao X. (2019). Targeted and effective glioblastoma therapy via aptamer-modified tetrahedral framework nucleic acid-paclitaxel nanoconjugates that can pass the blood brain barrier. Nanomedicine 21, 102061. 10.1016/j.nano.2019.102061 31344499

[B20] Shi Y. Jiang Y. Cao J. Yang W. Zhang J. Meng F. (2018). Boosting RNAi therapy for orthotopic glioblastoma with nontoxic brain-targeting chimaeric polymersomes. J. Control. Release 292, 163–171. 10.1016/j.jconrel.2018.10.034 30408555

[B21] Tamura R. Miyoshi H. Yoshida K. Okano H. Toda M. (2019). Recent progress in the research of suicide gene therapy for malignant glioma. Neurosurg. Rev. 44 (1), 29–49. 10.1007/s10143-019-01203-3 31781985

[B22] Tomar V. S. Patil V. Somasundaram K. (2020). Temozolomide induces activation of wnt/β-catenin signaling in glioma cells via PI3K/akt pathway: Implications in glioma therapy. Cell Biol. Toxicol. 36 (3), 273–278. 10.1007/s10565-019-09502-7 31758290

[B23] Tortorella S. Karagiannis T. C. (2014). Transferrin receptor-mediated endocytosis: A useful target for cancer therapy. J. Membr. Biol. 247 (4), 291–307. 10.1007/s00232-014-9637-0 24573305

[B24] Verheul T. C. J. van Hijfte L. Perenthaler E. Barakat T. S. (2020). The why of YY1: Mechanisms of transcriptional regulation by Yin Yang 1. Front. Cell Dev. Biol. 8, 592164. 10.3389/fcell.2020.592164 33102493 PMC7554316

[B25] Xiao B. Viennois E. Chen Q. Wang L. Han M. K. Zhang Y. (2018). Silencing of intestinal glycoprotein CD98 by orally targeted nanoparticles enhances chemosensitization of colon cancer. ACS Nano 12 (6), 5253–5265. 10.1021/acsnano.7b08499 29860836

[B26] Xie Y. Hang Y. Wang Y. Sleightholm R. Prajapati D. R. Bader J. (2020). Stromal modulation and treatment of metastatic pancreatic cancer with local intraperitoneal triple miRNA/siRNA nanotherapy. ACS Nano 14 (1), 255–271. 10.1021/acsnano.9b03978 31927946 PMC7041410

[B27] Yang J. Luo S. Zhang J. Yu T. Fu Z. Zheng Y. (2021). Exosome-mediated delivery of antisense oligonucleotides targeting α-synuclein ameliorates the pathology in a mouse model of Parkinson's disease. Neurobiol. Dis. 148, 105218. 10.1016/j.nbd.2020.105218 33296726

[B28] Yang K. Wu Z. Zhang H. Zhang N. Wu W. Wang Z. (2022). Glioma targeted therapy: Insight into future of molecular approaches. Mol. Cancer 21 (1), 39. 10.1186/s12943-022-01513-z 35135556 PMC8822752

[B29] Yasaswi P. S. Shetty K. Yadav K. S. (2021). Temozolomide nano enabled medicine: Promises made by the nanocarriers in glioblastoma therapy. J. Control. Release 336, 549–571. 10.1016/j.jconrel.2021.07.003 34229001

[B30] Yu X. Bai Y. Han B. Ju M. Tang T. Shen L. (2022). Extracellular vesicle‐mediated delivery of circDYM alleviates CUS‐induced depressive‐like behaviours. J. Extracell. Vesicles 11 (1), e12185. 10.1002/jev2.12185 35029057 PMC8758833

[B31] Zhao Y. Jiang Y. Lv W. Wang Z. Lv L. Wang B. (2016). Dual targeted nanocarrier for brain ischemic stroke treatment. J. Control. Release 233, 64–71. 10.1016/j.jconrel.2016.04.038 27142584

